# Complete Remission of a Refractory Acute Myeloid Leukemia with Myelodysplastic- and Monosomy 7-Related Changes after a Combined Conditioning Regimen of Plerixafor, Cytarabine and Melphalan in a 4-Year-Old Boy: A Case Report and Review of Literature

**DOI:** 10.3390/cancers10090291

**Published:** 2018-08-27

**Authors:** Antonio G. Grasso, Marilena Granzotto, Davide Zanon, Alessandra Maestro, Stefano Loiacono, Natalia Maximova

**Affiliations:** 1 Department of Medicine, Surgery and Health Sciences, University of Trieste, Piazzale Europa 1, 34127 Trieste, Italy; antonioggrasso@gmail.com; 2 Department of Laboratory Medicine, ASUITS, Piazza dell’Ospitale 1, 34129 Trieste, Italy; marilena.granzotto@asuits.sanita.fvg.it; 3 Pharmacy and Clinical Pharmacology Unit, Institute for Maternal and Child Health-IRCCS Burlo Garofolo, via dell’Istria 65/1, 34137 Trieste, Italy; davide.zanon@burlo.trieste.it (D.Z.); alessandra.maestro@burlo.trieste.it (A.M.); stefano.loiacono@burlo.trieste.it (S.L.); 4 Bone Marrow Transplant Unit, Institute for Maternal and Child Health-IRCCS Burlo Garofolo, via dell’Istria 65/1, 34137 Trieste, Italy

**Keywords:** acute myeloid leukemia, refractory, monosomy 7, pediatric, myelodysplastic, plerixafor, melphalan

## Abstract

Acute myeloid leukemia with myelodysplastic changes and monosomy 7 is a rare form of pediatric leukemia associated with very poor disease-free survival. The refractoriness of the disease is due to the protection offered by the bone marrow niche, making leukemic stem cells impervious to whatever chemotherapy or myeloablative regimen is chosen. Using a mobilizing agent for haematopoietic stem cells, Plerixafor, could sensitise leukemic cells to the myeloablative therapy. This approach was not previously used in a pediatric population, and in adult populations, was used in combination with busulphan with no difference in overall survival. We describe the case of a 4-year-old boy affected by refractory acute myeloid leukemia with myelodysplastic changes and monosomy 7. The child had never achieved a remission. We proposed a combined time-scheduled scheme of therapy with plerixafor and melphalan. Combining pharmacokinetics of plerixafor with pharmacokinetics and rapid and elevated myeloablative potential of melphalan in high dosage (200 mg/m^2^), we succeeded in mobilizing more than 85% of stem blasts immediately before infusion of Melphalan. The count of residual blasts after 8 h from melphalan infusion was only 1.3 cells/μL. The child achieved an engraftment at day +32 with full donor chimerism. Sixteen months after haematopoietic stem cell transplantation (HSCT), he is well and in complete remission. Our case suggests that the use of plerixafor before a conditioning therapy with melphalan could induce remission in acute myeloid leukemia refractory to the usual conditioning therapy in pediatric patients. This work adds strength to the body of knowledge regarding the “personalized” conditioning regimen for high-risk leukemic patients.

## 1. Introduction

Acute myeloid leukemia (AML) in children is rare in comparison with acute lymphoid leukemia, accounting for 10–15% [1] of cases of pediatric leukemia with an overall survival of almost 75% at five years [2]. However, the assessment of the cytogenetic and molecular features is essential for risk stratification in myeloid malignancies, AML included. All patients with high-risk characteristics need to achieve remission and undergo bone marrow transplantation [3], with an overall survival of 70% for those undergoing transplantation after complete remission and 50% for those undergoing transplantation after incomplete remission [4].

Molecular features of high-risk AML are translocation t(6;9) (p23;q34), monosomy/deletion of chromosome 5, mutation or duplication of FLT3 gene and monosomy/deletion of chromosome 7. The complete monosomy of chromosome 7 may be found in myeloid diseases as myelodysplastic syndrome and AML. In AML, although rare (5%), it is one of the severest and most conspicuous cytogenetic abnormalities, with an overall survival at 5 years null without haematopoietic stem cell transplantation (HSCT) and 35–50% with transplantation after achieving remission [5]. Even a matched unrelated donor transplant can offer a better survival compared with chemotherapy. However, achieving remission is crucial: undergoing transplantation without complete remission has an overall survival of 10–20%. Strategies to achieve remission and prevent post-transplant relapse are needed, but resistance to a regimen of chemotherapy is not uncommon [6].

We propose a combined conditioning approach using cytarabine (ARA-C), plerixafor, melphalan (L-PAM) as myeloablative and immunosuppressive therapy prior to HSCT. Plerixafor is an inhibitor of chemokine receptor type 4 (CXCR-4) and is commonly used to induce mobilization of haematopoietic stem cells (HSC) for apheresis prior to autologous HSCT. We assume that Plerixafor could induce susceptibility to conditioning therapy and report a pediatric case of a monosomy 7-related AML that was refractory to chemotherapy and standard conditioning but that had a complete molecular remission one year after the HSCT using this approach. 

## 2. Case Presentation 

We present the case of a 4-year-old boy with onset of AML with fever, abdominal pain, a very enlarged spleen palpable on transverse umbilical line, and hyperleukocytosis. The first evaluation of disease revealed a massive invasion of the bone marrow (50% of myeloid leukemia cells) with absence of invasion of the central nervous system. The karyotype analysis revealed a monosomy of chromosome 7 without translocations of prognostic impact at fluorescent in situ hybridization analysis. The child was therefore treated with European protocol LAM 2013/01 but showed no response to induction treatment with the persistence of 30% of blast cells at bone marrow aspiration. The bone biopsy, after induction phase, showed dysmorphic and dysplastic precursor myeloid cells of the three lineages, allowing diagnosis of acute myeloid leukemia with myelodysplasia-related changes (AML-MDC). Resistance to induction, monosomy 7 and the condition of AML-MDC categorize the disease as a high-risk AML, requiring the achievement of remission and bone marrow transplantation. The boy received two cycles of idarubicin, cytarabine, and etoposide and one cycle of fludarabine and high-dose cytarabine as salvage therapy without response. At the end of therapy, peripheral blood immunophenotypic analysis showed a persistence of CD34+ CD117+ CD33− blast cells (14% of leukocytes, 435 cell/μL). Because of the disease severity, we chose to continue the program of HSCT and started conditioning treatment.

**Figure 1:**
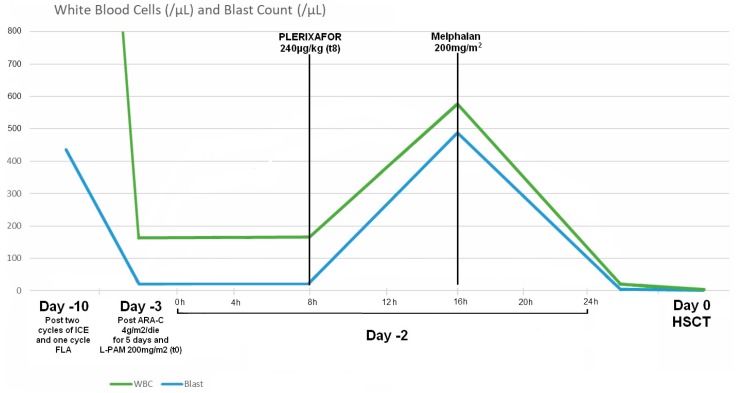
Blast cell count and White Blood Cell count after salvage therapy (Day −10), after conditioning (Day −3 t0), after administration of plerixafor (Day −2 t8) and after administration of melphalan (Day −2 t16) and immediately before of haematopoietic stem cell transplantation (HSCT) (Day 0).

The boy received high doses of ARA-C (4 g/m^2^/die) for 5 days prior to transplant on day 0, L-PAM (200 mg/m^2^) and antithymocyte globulin (6.3 mg/kg/die for three days). Despite the high-dose ARA-C treatment, at day −3, peripheral blood flow cytometric analysis still showed presence of blast cells (13% of leukocytes; 21 blast cells/µL). At day −2, we administered a dose of 240 µg/kg of plerixafor, an inhibitor of CXCR4, to mobilize staminal CD34+ leukemic cells from bone marrow. After 8 h and 1/2 from the subcutaneous injection of plerixafor, we analyzed all CD34+ cells in peripheral blood, finding the expected massive mobilizing effect with 493 CD34+ cells/μL (85% of blast cells on peripheral white blood count). At the same time, we started the administration of L-PAM 200 mg/m^2^ in an hour. The conditioning regimen was well tolerated without any adverse effect (Figure 1).

**Figure 2:**
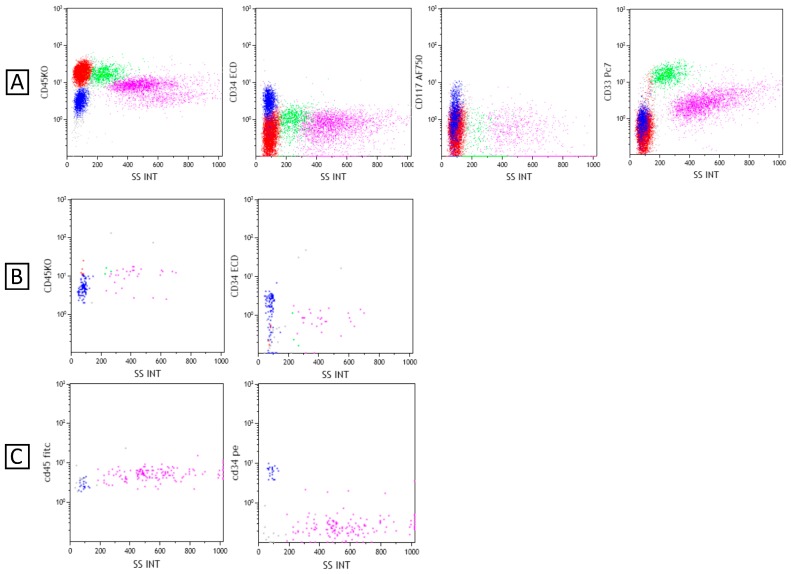
Flow cytometric analysis of peripheral blood cells revealed blast cells (blue dots) positive for CD34, CD117 (heterogeneous expression) and negative for CD33 at diagnosis (**A**). After 8 h from the administration of Plerixafor the absolute count of blast cells rose to 485/μL with a white blood count of 570/μL (**B**). After 8 h from the administration of L-PAM, the blast cell absolute number was of 1.3 cells/μL and there was a very low overall white blood count of 20 cells/μL (**C**).

Restudying the peripheral blood cells by immunophenotyping, after 8 h from the administration of L-PAM, we found a blast cell absolute number of 1.3 cells/μL and a very low overall white blood cell count of 20 cells/μL (Figure 2). On day 0, before transplant, we did not find any cells identifiable with flow cytometric analysis and with qPCR. 

We infused 6.6 × 10^8^ total nuclear cells/kg from the 10/10 HLA matched unrelated donor, AB0 mismatched. Tacrolimus and mycophenolate mofetil were used as graft-versus-host disease (GVHD) prophylaxis. An engraftment was successfully achieved, confirmed by peripheral blood full donor chimerism analysis at day +32. 

During the engraftment, we monitored minimal residual disease (MRD) with flow cytometric analysis without finding any statistically significant sign of relapse. We outlined a protocol to detect simultaneous expression of several markers (CD34, CD33, CD117, CD13, HLADR, CD11b, CD16, CD66b, CD14, CD45) on AML blast cells by multiparametric flow cytometry. The combination of these markers can monitor MRD at a sensitivity of 10^−4^. 

The post-transplant period was complicated by early and immediately severe GVHD grade IV that necessitated a combined therapy with methylprednisolone (3 mg/kg) and one dose of methotrexate (13 mg/m^2^) without a significant response. Intestinal damage was still symptomatic, causing a diffuse thickening of the ileum and colic walls with mesenteric herniation at the abdomen ultrasonography. Because of GVHD steroid refractoriness, the boy was treated with anti-Tumor necrosis factor-α therapy with infliximab (10 mg/kg/dose weekly) with a good response immediately after the first administration. The cutaneous symptoms still remained only partially controlled and, due to a new serious reactivation, on day +41 the patient received a single dose of fludarabine with a progressive and stable remission of the signs of disease. 

During the post-transplant period, the boy was affected by a systemic infection caused by human herpesvirus 6 and cytomegalovirus and by a BK polyomavirus-related hemorrhagic cystitis, without any consequences and a good response to the therapy. Liver biopsy confirmed a mild cholestatic hepatitis due to almost complete ductopenia, which was treated with ursodesossicolic acid. After 12 months from transplant, the boy was still in complete remission, validated by both the bone biopsy, which showed only a hypocellular bone marrow with a normal myeloid series, and the immunophenotypic and MRD analyses of bone marrow cells, which showed complete remission. With no severe morbidity and a complete engraftment with a normal function of B-cells, the boy was able to return to the vaccination calendar with inactivated vaccines.

Informed consent for publication was obtained and is available for review by the Editor.

## 3. Results and Discussion 

Monosomy 7-related AML is one of the most refractory leukemia diseases. Although it is possible to achieve remission with induction (62%), post-transplant relapse is common and involves almost 80% of treated AML, of which 75% occur in the first year after transplantation. In comparison, the overall incidence of AML relapses is 25–35%, with a worse prognosis for those occurring in the first year [7,8].

One of the resistance mechanisms of leukemia cells is finding “sanctuaries” where chemotherapy cannot induce its pro-apoptotic and toxic effects. Current evidence suggests that the molecular features of staminal leukemic cells change the interaction between the bone marrow niche microenvironment and normal cells, developing resistance to common treatment regimens. The bone marrow niche influences survival and development of normal stem cells, and staminal leukemia cells enhance and overexpress proximity and soluble involved factors [9,10]. One of the most important mechanisms involved is the binding between CXCR4 on the endothelial cells and its ligand C-X-C chemokine receptor 12 (CXC12 or SDF1) on the stromal structure of the niche, which induce crucial pathways for stem cell survival and homing in bone marrow [11,12]. The exposition of CXCR4 in myeloid leukemia cells is associated with a very poor prognosis, confirming the idea that the niche has a protective function against chemotherapy for leukemia cells [13,14].

We postulated that using Plerixafor, a CXCR4 inhibitor, could induce staminal leukemia cells to mobilize in the bloodstream, making them vulnerable to a conditioning regimen, and so achieving remission and letting the patient undergo the bone marrow transplantation. The mobilizing effect is mediated by the inhibition of the molecular binding between CXCR4 and CXC12, releasing CD34+ cells into the bloodstream and interfering with the survivor’s downstream pathway (such as PI3K/AKT) [15]. 

Chambon et al. [16] showed that in children in a ‘one-day’ mobilization with a dosage of 240µg/kg, the maximum CD34+ peak was reached between 4 and 7 h after plerixafor. Maschan et al. [17] obtained a successful mobilization with plerixafor in 31 patients without relevant toxicity after 11 h from injection. Liles et al. [18] obtained the best dose-response at 9 h after administration of 240 µg/kg of plerixafor. Poor mobilizers seem to reach the peak earlier than good mobilizers, in a period between 3 and 6 h: this parameter could be very important in monitoring CD34+ release and, for this reason, we started the infusion of melphalan only after monitoring the HSC count in peripheral blood [19,20]. All the analyzed studies showed a progressive reduction of CD34+ count around three hours after the peak.

Key to maximizing the antileukemia cells’ effect is, in our theory, administering the conditioning regimen at the exact moment when the mobilizing effect on leukemia cells of plerixafor is maximum. To achieve this effect, the myeloablative regimen was chosen after an accurate analysis of the pharmacokinetics of melphalan and busulphan. The latter shows a wide interpatient variability due principally to its variable hepatic metabolisms, but also linked to age and clearance. Furthermore, busulfan is given on multiple days in the conditioning regimens and has steady myeloablative effects. Animal models show an effect on CD34+ cells inducing senescence but not apoptosis after the intravenous infusion, with only a progressive ablation of the haematopoietic stem cells [21,22,23,24]. Unlike busulfan, L-PAM has a rapid and immediate action against leukemia cells. L-PAM has a half-life of 90 min ± 57 and this short period is linked to its high level of spontaneous degradation and renal excretion. A high dose of melphalan, such as 200 mg/m^2^, could possibly lead to a change in its kinetics, prolonging its half-life [25]. 

Plerixafor administration prior to conditioning or chemotherapy regimen [26] was already tried in adults affected by AML, using a combined regimen of busulfan and cytarabine, but the results, despite showing a high success of complete remission prior to HSCT, showed no difference in survival and relapse compared with the traditionally treated cohort without plerixafor [27]. Both studies used granulocyte colony stimulating factors to enhance the effect of plerixafor but, paradoxically, as the authors speculate, combined with chemotherapy, the prosurvival effect of the drug on HSC could counteract the pro-apoptotic mobilizing effect of the plerixafor. 

Finally, we observed a late engraftment and a massive acute GVHD in our patient that was resistant to the standard immunosuppressive therapy and responded to fludarabine only. This is in contrast with other studies involving plerixafor, where severity and incidence of GVHD was lower in comparison with the historical cohort [27]. We could assume that the massive myeloablative effect of L-PAM on a mobilized bone marrow disrupts even the little quota of chimerism that leads to an equilibrium between graft and host, altering the structure of the niche inhibiting CXCR4, slowing the engraftment and lowering the action of T-Reg cells whose action is mediated by interaction between CXCR4 and CXC12 [28]. The massive acute GVHD may have helped achieve remission by inducing a graft-versus-leukemia effect (GVL), but data from historical cohorts are not clear about the role of GVL in refractory disease and do not show any correlation of the incidence of relapse with the grade of acute GVHD in AML with or without myelodysplastic changes, unlike the proven correlation in acute lymphoblastic leukemia [29,30]. We could speculate that GVL alone could not induce remission in refractory disease and requires the prior sensitization and cytoreductive effects of the plerixafor–melphalan combination to achieve its antileukemic effect. 

## 4. Conclusions

In conclusion, we would highlight some considerations from our case: first of all, the inhibition of CXCR-4 also mobilizes staminal leukemia cells that may be found in peripheral blood; second, our hypothesis is that chemotherapy and myeloablative agents could be more effective if used in combination with “chemotherapy-sensitising” agents, taking into account the timing of the maximal effect of the two drugs. Lastly, this approach could also achieve remission in refractory/relapsed disease that normally almost always has a precocious relapse between one month and six months after transplantation. This is the first described pediatric case where plerixafor was used combined with a myeloablative regimen to achieve remission in leukemia, and the first in either a pediatric or adult population using L-PAM.
